# RETRACTION: A Peptide Analogue of Selectin Ligands Attenuated Atherosclerosis by Inhibiting Monocyte Activation

**DOI:** 10.1155/mi/9763878

**Published:** 2025-12-10

**Authors:** Mediators of Inflammation

RETRACTION: Z. Ye, S. Zhang, Y. Liu, S. Wang, J. Zhang, and R. Huang, “A Peptide Analogue of Selectin Ligands Attenuated Atherosclerosis by Inhibiting Monocyte Activation,” *Mediators of Inflammation* no. 2019 (2019). https://doi.org/10.1155/2019/8709583.

The above article, published online on 6 May 2019 in Wiley Online Library (wileyonlinelibrary.com), has been retracted following the agreement of the journal’s Chief Editor, Anshu Agrawal; and John Wiley & Sons Ltd.

The retraction has been agreed following an investigation of the concerns raised by a third party, which identified multiple instances of inappropriately overlapping figures. More specifically:

- The P‐Sel+/IELLQAR+ panel of Figure 2c is identical to the Control panel of Figure 2e of [1].
- The P‐Sel+/ICAM‐1+/IELLQAR+ panel of 2c is overlapping with both the P‐Sel+/ICAM+ panel of Figure 2d and the LPS panel of Figure 2e in [1].
- The P‐Sel+ panel of 2d is overlapping with both the P‐Sel+/IACM‐1+/IELLQAR+ panel of Figure 2d and the P‐Selectin panel of Figure 2e in [1].
- In Figure 2e, the fluorescence image of the monocytes in the PBS /Huvec (PBS) panel of the PBS group shares an unexpected similarity with the monocytes from the P‐sel+IELLQAR /Huvec (PBS) group in Figure 2f.
- In Figure 7, the Western Blot bands corresponding to PI3K P85 are identical for both monocyte types shown in the figure, despite being obtained under different conditions.

As a result of our investigation, the conclusions of the article can no longer be considered reliable and it is therefore retracted.

The authors have been informed of this retraction but did not provide a response.
